# Sex identification of sun conure (*Aratinga solstitialis*) using loop-mediated isothermal amplification of W and Z spindlin chromosomes

**DOI:** 10.14202/vetworld.2024.2000-2007

**Published:** 2024-09-08

**Authors:** Parichart Wancham, Sakuna Phatthanakunanan, Siriluk Jala, Kanyakodchanan Woramahatthanon, Supaphen Sripiboon, Preeda Lertwatcharasarakul

**Affiliations:** 1 Animal Health and Biomedical Sciences Study Program, Faculty of Veterinary Medicine, Kasetsart University, Bangkok 10900, Thailand; 2 Kamphaeng Saen Veterinary Diagnostic Center, Faculty of Veterinary Medicine, Kasetsart University, Nakorn Pathom 73140, Thailand; 3 Department of Large Animal and Wildlife Clinical Science, Kasetsart University, Nakorn Pathom 73140, Thailand; 4 Department of Pathology, Kasetsart University, Nakorn Pathom 73140, Thailand

**Keywords:** loop-mediated isothermal amplification, sex identification, spindlin gene, sun conure

## Abstract

**Background and Aim:**

The sun conure (*Aratinga solstitialis*), a bird belonging to the Psittaciformes family, is a popular pet because of its bright color and beautiful appearance. The sun conure is a monomorphic bird with similar appearances between males and females, making sex identification difficult by observing the external morphology. Therefore, molecular techniques are utilized. Loop-mediated isothermal amplification (LAMP) is a molecular technique that is often applied for sex identification in birds and is a quick and simple method that can be used in the field. This study used the LAMP technique to improve sex identification in sun conures by observing the color change of hydroxy naphthol blue.

**Materials and Methods:**

Two primer sets, SunSpin-W and SunSpin-Z, were designed for sex identification in sun conures using the LAMP technique specific to the spindlin gene. The developed LAMP reaction was tested for optimal conditions, sensitivity, and specificity compared with the polymerase chain reaction (PCR) technique.

**Results:**

The SunSpin-W primer set amplified only female birds, whereas the SunSpin-Z primer set amplified DNA from both male and female birds. The primer sets were optimized at 62°C for 45 min. A positive result was visible to the naked eye from the color change of the reaction. In the LAMP assay, the lowest detectable concentration was 10 pg/μL and in the PCR assay, it was 1 ng/μL, while a 100% accuracy rate in sex identification was observed when comparing the LAMP assay results with the PCR assay.

**Conclusion:**

This study successfully developed a LAMP technique for sex identification of sun conure, which took 45 min to complete and can be expanded for use in the field.

## Introduction

Sun conures (*Aratinga solstitialis*), birds belonging to the Psittacidae family, are popular pets and are valued because of their vibrantly colored feathers, playful personality, and friendliness. Because of their popularity, sun conures are often bred for commercial purposes. A key component of successful breeding is accurate sex identification between males and females. However, the sun conure is a monomorphic bird with similar male and female appearances, making sex identification by external morphology [1]. There is a high demand for sun conures, and unsuccessful breeding may lead to illegal catches and importation.

Avian sex identification based on molecular techniques is now widely used to differentiate the heterogametic (ZW) and homogametic (ZZ) sex chromosomes of male and female birds [2]. In Psittacidae, the main molecular technique for sex identification is polymerase chain reaction (PCR) based on sex-limited markers [3]. Many genetic sex markers have been suggested for PCR-based molecular sexing, such as chromo-helicase-DNA binding protein 1 [4], the Nipped-B homolog gene [5], the RAS p21 protein activator 1 gene (RASA1) [6], and the 0.6 kb *Eco*RI fragment (EE 0.6) [7], as well as the spindlin gene in birds that exhibit different nucleotide sequences on the W and Z chromosomes [8]. In this study, the spindlin gene was effectively utilized for sex identification in Psittacidae [9].

The loop-mediated isothermal amplification (LAMP) technique has accuracy similar to PCR with a reaction time. It does not require electrophoresis for the interpretation of results [10]. LAMP DNA amplification reactions can be detected by monitoring turbidity [11], the fluorescence of phosphor under ultraviolet (UV) light [12, 13], and visual color changes using color indicators to simplify the interpretation of results [14, 15]. Collectively, these features make LAMP a convenient, user-friendly, and time-efficient technique. Therefore, this study developed the LAMP assay using a newly designed primer set based on the spindlin gene to provide a sex identification method for sun conure, which is a completely new primer design that can be developed for use in the field. It reduces problems related to time and correct breeding pairing.

## Materials and Methods

### Ethics approval

All animal experiment protocols were approved by the Institutional Laboratory Animal Care and Use Committee of the Kasetsart University of Thailand (Approval number ACKU65-VET-069, September 12, 2022).

### Study period and location

The study was conducted from August 2021 to May 2023 at the Biotechnology Laboratory, Kamphaeng Saen Veterinary Diagnostic Center, Faculty of Veterinary Medicine, Kasetsart University, Kamphaeng Saen Campus.

### Sample collection

In total, 94 dried blood spot samples from sun conures were obtained from the Kamphaeng Saen Veterinary Diagnostic Center. Each sample was stored in a zipper bag at –20°C until DNA extraction.

### DNA extraction

DNA was extracted from dried blood spots using a modified alkaline extraction method [16]. Approximately 3 × 3 mm of dried blood spots were placed in a sterile microtube, added with 20 μL of 0.2 M NaOH, and incubated at 75°C for 20 min. Then, 180 μL of 0.05 M Tris-HCl was added and mixed by a vortex. The extracted DNA was stored at –20°C until use.

### LAMP primer design

A LAMP primer set of each spindlin W and Z gene was designed using the NEB LAMP Primer Design Tool (https://lamp.neb.com/#!/) based on the nucleotide alignment between the spindlin-W gene (accession no. XM_030510990 and XM_030510991) and the spindlin-Z gene (accession no. XM_030471366 and XM_030471367) from the GenBank database using the ClustalW program in BioEdit version 5.0.9 software. Each primer set consisted of six primers: forward outer primers (F3), backward outer primers (B3), forward internal primers (FIP), backward internal primers (BIP), forward loop primers, and backward loop primers.

### Spindlin-gene cloning

DNA extracted from male and female sun conures was amplified for spindlin using PCR with the F3/B3 primers of each set. Amplicons of both sexes were cut from agarose gels and purified using a FavorPrep™ GEL/PCR Purification Mini kit (Favorgen Biotech Corporation, Taiwan) according to the manufacturer’s instructions. The purified amplicons were cloned in a pLUG-Prime® TA-cloning Vector Kit II (iNtRON Biotechnology, Inc., Korea) and transformed into the *Escherichia coli* JM109 strain. PCR was performed to identify bacteria containing the target gene. Plasmids from bacteria cultured in LB broth overnight were extracted using a FavorPrep™ Plasmid Extraction Mini Kit (Favorgen Biotech Corporation) following the manufacturer’s recommendation and used to optimize the LAMP reaction conditions.

### Optimization of the LAMP reaction conditions

A T100™ Thermal Cycler (Bio-Rad, USA) was used to optimize the LAMP reaction. The volume of the LAMP reaction was 25 μL consisting of 1.4 mM deoxynucleotide triphosphates (dNTPs), 1X Isothermal Amplification Buffer, 1 μL of 8 U of *Bst* 2.0 WarmStart DNA polymerase (New England Biolabs, USA), and six primers: 1.6 μM inner primers (FIP and BIP), 0.4 μM outer primers (F3 and B3), 1 μM loop primers (LF and LB), 10 mM Magnesium sulfate (MgSO_4_), 1 M betaine, 0.16 mM hydroxy naphthol blue (HNB) (Loba Chemie Laboratory Reagents and Fine Chemicals, India), and 5 μL plasmid DNA template and the reaction was adjusted to a volume of 25 μL with nuclease-free water. All parameters relating to the optimal conditions of the LAMP assay were tested, including the differences in amplification temperatures (between 55°C and 65°C), the concentration of MgSO_4_ (6–12 mM), the concentration of betaine (0.2–1 M), and reaction times (15, 30, 45, and 60 min).

### PCR

The PCR reaction was prepared in a volume of 25 μL consisting of DreamTaq™ Green PCR Master Mix (2X) (Thermo Fisher Scientific, USA), 0.4 mM of each primer (2550F and 2718R) previously reported by Fridolfsson and Ellergen [17], and DNA template 5 μL. The reaction volume was adjusted to a volume of 25 μL with nuclease-free water. The reaction conditions consisted of pre-denaturation at 94°C for 5 min followed by 35 cycles of 94°C for 30 s, 57°C for 30 s, and 72°C for 30 s, with a final extension at 72°C for 5 min. The PCR products (5 μL) were then analyzed using 1.5% agarose gel electrophoresis with tris-acetate-ethylenediaminetetraacetic acid (TAE) buffer.

### Sensitivity and specificity of the LAMP assay

DNA concentrations were measured using a NanoDrop™ 2000 Spectrophotometer (Thermo Fisher Scientific). To determine the sensitivity of the assay, 10-fold serial dilutions of a DNA concentration of 10 ng/μL to 100 fg/μL were used as DNA templates. The LAMP reaction was performed under the optimized conditions, and 5 μL of the product was analyzed using 1.5% agarose gel electrophoresis and observed visually from the LAMP reaction tube. If the reaction occurred, the solution color changed from purple to sky blue and remained purple in the absence of the reaction.

The specificity of the LAMP reaction was assessed using DNA extracted from 94 blood samples consisting of 47 females and 47 males, which were sex-identified by PCR using the primers 2550F/2718R [17]. The DNA of seven different avian species, including African gray (*Psittacus Erithacus*), Cockatiel (*Nymphicus hollandicus*), Eclectus (*Eclectus roratus*), Forpus (*Forpus* spp.), Lovebird (*Agapornis* spp.), and Rose-ringed parakeet (*Psittacula Kramer*), was also tested.

## Results

**Figure-1:**
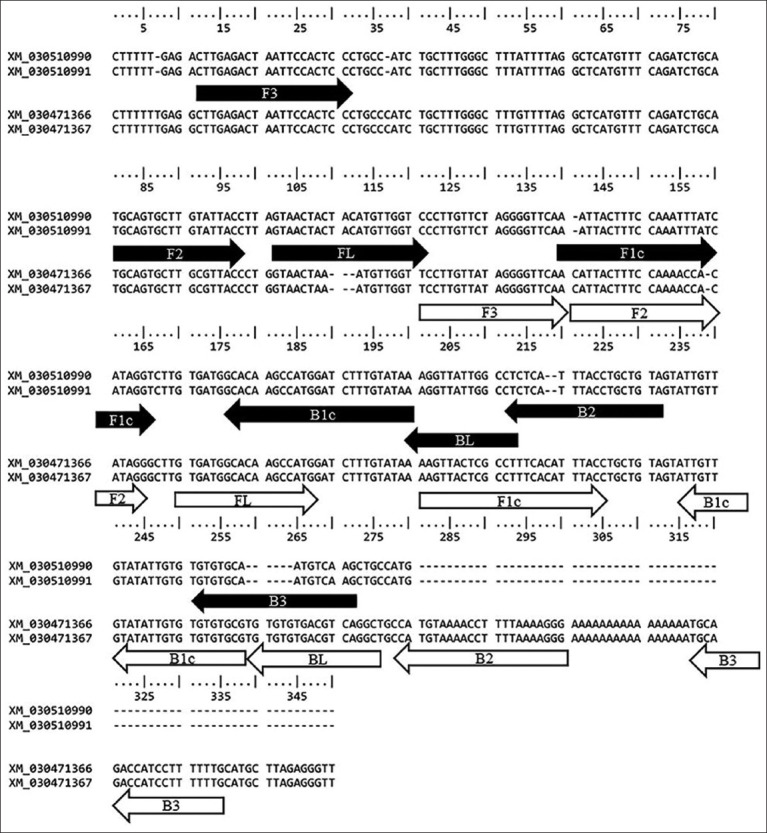
Multiple nucleotide alignment of spindlin on the sex chromosome and the location of the designed primers. (Black arrow is primer set SunSpin-W and white arrow is primer set SunSpin-Z).

SunSpin-W and SunSpin-Z primer sets were designed explicitly for the spindlin gene of W and Z chromosomes, respectively. Each primer set comprised six primers encompassing eight specific regions (Figure-1). The oligonucleotide sequences of the primers are summarized in Table-1.

**Table-1:** Information of two LAMP primer sets for recognizing the spindlin genes on the W and Z chromosomes.

Primer set	Primer	Oligonucleotide (5′→3′)
SunSpin-W	F3	CTTGAGACTAATTCCACTCCC
	B3	CTTGACATCATGCACACA
	FIP	ACCTATGATAAATTTGGAAAGTAATTATGCAGTGCTTGTATTACC
	BIP	GCACAAGCCATGGATCTTTGTATAAAATACTACAGCAGGTAAATGAGAG
	FL	GACCAACATGTAGTAGTTAC
	BL	AAAGGTTATTGGCCT
SunSpin-Z	F3	TCCTTGTTATAGGGGTTCAA
	B3	CAAAAAAGGATGGTCTGCA
	FIP	GGTAAATGTGAAAGGCGAGTAACTTCATTACTTTCCAAAACCACATAGG
	BIP	ATTGTTGTATATTGTGTGTGTGCGCCCTTTTAAAAGGTTTTACATGG
	FL	CATGGCTTGTGCCATCAC
	BL	TGTGTGTGACGTCAGGC

LAMP=Loop-mediated isothermal amplification

**Figure-2:**
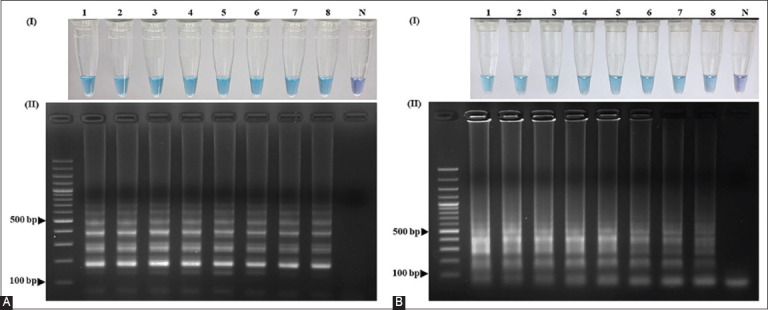
Optimization of the amplification temperatures for the LAMP primer sets (A) SunSpin-W and (B) SunSpin-Z. Visual inspection of amplified LAMP products with (I) HNB under daylight and (II) 1.5% agarose gel electrophoresis system. (N: non-template control, 1: 65°C, 2: 64.3°C, 3: 63°C, 4: 61.1°C, 5: 58.8°C, 6: 56.9°C, 7: 55.7°C, and 8: 55°C). LAMP=Loop-mediated isothermal amplification, HNB=Hydroxy naphthol blue.

In the LAMP assay, amplification reactions were performed at different temperatures ranging from 55°C–65°C. Results showed that both primer sets amplified the target gene at all temperatures with visualization of color changes (Figure-2I) and agarose gel electrophoresis (Figure-2II), whereas the temperature range of 58.8°C–65°C gave brighter bands in agarose gel electrophoresis than at other temperatures. A temperature of 62°C at which both sets of primers could be used for the sex identification of sun conure using the LAMP technique was chosen for the next experiment.

**Figure-3:**
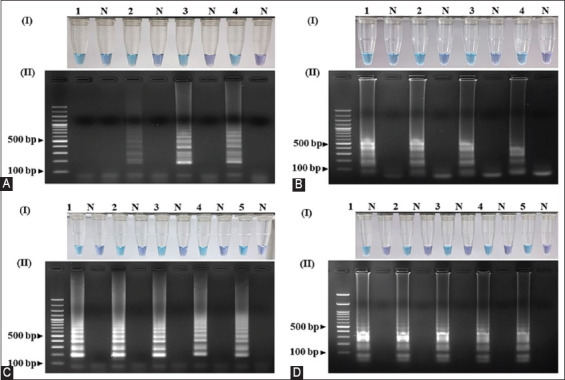
Optimization of the LAMP assay with different concentrations of MgSO_4_ using the LAMP sexing primer sets (A) SunSpin-W and (B) SunSpin-Z. (N: non-template control, 1: 6 mM, 2: 8 mM, 3: 10 mM, and 4: 12 mM). Optimization of the LAMP assay with different concentrations of betaine in the LAMP sexing primer sets (C) SunSpin-W and (D) SunSpin-Z. (N: Non-template control, 1: 0.2 M, 2: 0.4 M, 3: 0.6 M, 4: 0.8 M, and 5: 1 M). Visual inspection of amplified LAMP products with (I) HNB under daylight and (II) 1.5% agarose gel electrophoresis system. LAMP=Loop-mediated isothermal amplification, HNB=Hydroxy naphthol blue.

To convert the color from purple to sky blue, the optimal MgSO_4_ concentrations for the SunSpin-W primer set were 10–12 mM (Figure-3AI), whereas at 10 mM, the electrophoresis amplicons were brighter (Figure-3AII). A MgSO_4_ concentration of 6–12 mM for the SunSpin-Z primer caused the color of the sample to shift from purple to sky blue (Figure-3BI), and a DNA band appeared in the agarose gel electrophoresis (Figure-3BII). Because the color change was visible, both primer sets were used to identify the sex of the sun conures using the LAMP technique, and a concentration of 10 mM was selected. Amplification was detected at all betaine concentrations in both primer sets; hence, 0.2 M was selected as the final concentration for optimization (Figures-3C and D).

**Figure-4:**
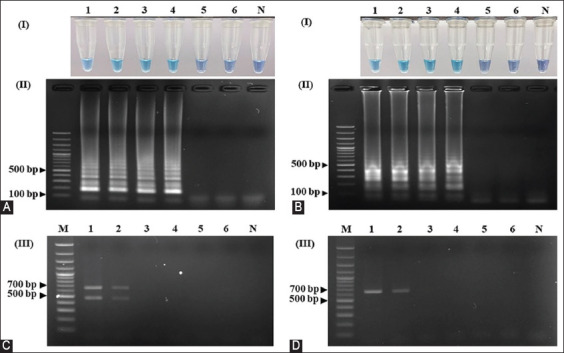
Limit of detections of the LAMP and PCR techniques. The LAMP technique using the (A) SunSpin-W and (B) SunSpin-Z primer sets was detected by (I) naked eye visual and (II) 1.5% agarose gel electrophoresis. PCR using 2550F/2718R detects extracted DNA from (C) female and (D) male (Lane M: DNA ladder, N: non-template control, 1: 10 ng/μL, 2: 1 ng/μL, 3: 100 pg/μL, 4: 10 pg/μL, 5: 1 pg/μL, and 6: 100 fg/μL). LAMP=Loop-mediated isothermal amplification.

**Figure-5:**
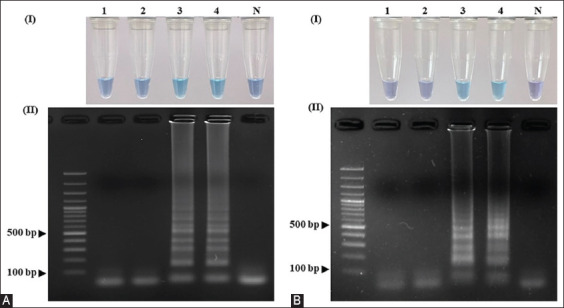
Reaction time optimization of LAMP to detect 10 pg/μL DNA template at 62°C using the primer sets (A) SunSpin-W and (B) SunSpin-Z. Visual inspection of the amplified LAMP products with (I) HNB under daylight and (II) 1.5% agarose gel electrophoresis system. (N: non-template control, 1: 15, 2: 30, 3: 45, and 4: 60 min). LAMP=Loop-mediated isothermal amplification.

The limit of detection (LOD) of the LAMP assays was compared with that of the PCR assay using sun conure DNA as a template. The LOD of the LAMP technique using both primer sets, SunSpin-W and SunSpin-Z, was 10 pg/μL, which was detected visually by the naked eye (Figures-4AI and BI) and by agarose gel electrophoresis (Figures-4AII and BII). When using the PCR technique, the lowest detectable levels were 1 ng/μL in both male and female birds (Figures-4CIII and DIII). LAMP assays to detect 10 pg/μL DNA templates were run at 15, 30, 45, and 60 min to determine the optimal assay duration. The observed color changes and clear gel electrophoresis results for both primer sets indicated that DNA target amplification in both primer sets required at least 45 min to occur (Figures-5A and B). Considering the above data, the optimal LAMP conditions for sex identification using the SunSpin-W and SunSpin-Z primer sets were 62°C with 10 mM MgSO_4_, 0.2 M betaine, and a reaction time of 45 min.

**Figure-6:**
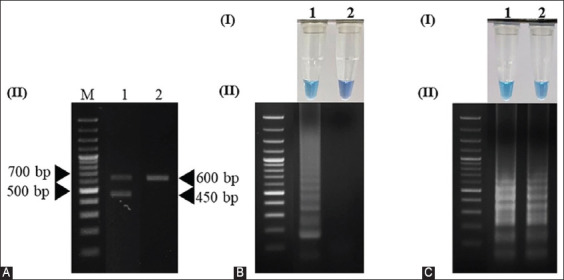
Comparison of sex identification results between PCR using (A) 2550F/2718R primer, LAMP techniques primers set (B) SunSpin-W and (C) SunSpin-Z. Visual inspection of amplified LAMP products with (I) HNB under daylight and (II) 1.5% agarose gel electrophoresis system. (Lane M: DNA ladder, 1: Female sun conure, 2: male sun conure). LAMP=Loop-mediated isothermal amplification, PCR=Polymerase chain reaction.

In a comparison of sex identification results between LAMP and PCR techniques, the PCR technique based on 2550F/2718R primers gave two bands (600 bp and 450 bp) in females and only one band (600 bp) in males (Figure-6A). In the case of the LAMP technique, the ladder-like band and color change appeared only in females and both sexes when using the SunSpin-W and SunSpin-Z primer sets, respectively (Figures-6B and C). A total of 94 sun conures (47 males and 47 females) were used to compare the efficiency of these techniques. We found that the results obtained by both methods were similar.

LAMP using SunSpin-W only amplified DNA samples from females, whereas LAMP using SunSpin-Z amplified DNA samples from both males and females. The LAMP technique was used for sex identification in seven different avian species. Results showed that these primer sets were able to identify the sex of other birds, including African gray (*P. Erithacus*), Cockatiel (*N. hollandicus*), Eclectus (*E. roratus*), Forpus (*Forpus* sp.), and Lovebird (*Agapornis* spp.), excluding the Rose-ringed parakeet (*P. Kramer*), which could not be sex-identified using this primer.

## Discussion

The CHD gene is a general genetic marker for identifying the sex of birds of different species. The CHD gene exhibits high conservation and distinct lengths on the Z and W chromosomes due to various intron sizes. Result interpretation usually depends on the number of bands identified by PCR, which requires electrophoresis to separate amplicon sizes. Female birds have two sizes of the CHD genes from one allele on the W and another allele on the Z CHD chromosome, whereas male birds have one size from two alleles on the Z CHD chromosome [17].

One of the few genes that is duplicated on both the W and Z chromosomes is the avian spindlin gene. This gene is useful for the sex identification of most bird species because of the divergence of intron sequences and the conservation of exon nucleotide sequences across at least 130 million years. The nucleotide sequences of the W- and Z-chromosome copies of this gene differ because the W chromosome does not undergo recombination. The third intron sequences of the Z-and W-chromosome spindlin genes were used to study the evolutionary relationships among certain Psittaciformes [9]. This study designed specific primer sets based on both the Z-and W-chromosome spindlin genes of birds using the LAMP technique. The results showed that LAMP separated Z- and W-chromosome spindlin genes and determined the identity of sun conure.

PCR is an accepted technique for identifying the sex of birds, but it has some significant drawbacks. Specialized laboratory equipment for temperature cycling control and gel electrophoresis for amplicon separation are required; hence, starting a new laboratory is difficult and inappropriate for fieldwork [18]. The high sensitivity and specificity of LAMP, together with the rapid and visual observability of the results, make this method suitable for detecting a variety of microorganisms without the need for a thermal machine and electrophoresis device [10]. The traditional LAMP reaction consists of four primers (F3, B3, FIP, and BIP) that recognize six target gene regions. Six designed primers were applied to eight positions of the spindlin gene. The reaction of the LAMP procedures was accelerated more than 100 times by two of the six primers, known as loop primers (LF and LB), which hybridized to the stem-loop area into the structures of the LAMP products [19].

MgSO_4_ is a cofactor that stimulates the activity of DNA polymerase and is responsible for binding to dNTP, primers, and DNA templates [20]. The most obvious color change was observed at 10 mM. The concentration of MgSO_4_ affects the specificity and the process of DNA amplification [21]. Two mechanisms of betaine in the LAMP process have been proposed: First, it prepares the DNA template for DNA polymerase [20], and second, it causes unstable GC-rich sequences that facilitate the separation of the template DNA strands [21]. From the experimental results, the optimized final concentration from 0.2 M to 1 M was not different in both primer sets, consistent with a previous study by Jeevalatha *et al*. [22] that tested a betaine concentration of 0.2 M–0.8 M and found that every concentration of betaine caused a LAMP reaction.

Visualizing the DNA products of the LAMP reaction on gel electrophoresis is not necessary, but it can be done by looking for turbidity, which is caused by the release of free pyrophosphates during DNA amplification [11]. Determining the turbidity of low-quality products is challenging. Under natural or UV light, SYBR green I, a fluorescent indicator that also requires post-amplification, was visible [23]. A drawback of this procedure is that adding SYBR green I requires opening the cover of the tube, which increases the risk of product spread. This study used HNB as an indicator, which was added to the reaction before the amplification began. Without opening the microtube, it was possible to see the purple color turn to sky-blue [24] with the naked eye, significantly reducing the risk of contamination.

Results showed that the LAMP assay had a 100-fold higher sensitivity than PCR. This finding is consistent with previous studies by Tang *et al*. [23], Naveen and Bhat [25], and Wang *et al*. [26], who reported that LAMP had up to 100 times higher detection sensitivity than PCR. The experimental results demonstrated that the sex identification of sun conures using the SunSpin-W and SunSpin-Z primer sets was 100% accurate and was also able to sex-identify other bird species in the Psittaciformes family using both primer sets. The accuracy of sex identification of sun conures using LAMP was comparable to that of PCR.

LAMP is a simple technique that uses fewer laboratory instruments. After target DNA amplification, the results can be visualized by the naked eye without the need for an open microtube, which reduces contamination of the amplicons in the working area [27, 28]. Therefore, the LAMP assay is a good choice for bird sex identification in the field.

## Conclusion

The LAMP technique for sex identification in sun conures and several other parrot species was developed by focusing on the spindlin gene. Color changes indicated positive results after 45 min of reaction time, and no complex procedures or usage-appropriate specialized equipment was required. Consequently, the proposed technique can enhance field usability and convenience. While our approach demonstrates promise, there are notable limitations that need addressing. Specifically, the method for sex identification requires the execution of two separate reactions (SunSpinW and SunSpinZ) from a single sample. To overcome this, future work should focus on enhancing the methodology to enable result analysis within a single tube.

## Authors’ contributions

PW and PL: Designed and supervised the study and drafted and revised the manuscript. SP and KW: Preparation of gene constructs and conducted the PCR. SJ: Optimization of the LAMP technique. SS: Supervised the study and revised the manuscript. All authors have read, reviewed, and approved the final manuscript.
